# Biomarkers for Necrotising Enterocolitis—Are We There Yet?

**DOI:** 10.3390/children13070894

**Published:** 2026-07-03

**Authors:** Anna Jackson, Maria Cifuentes Nino, Janet Berrington

**Affiliations:** 1 Newcastle Neonatal Services, Newcastle upon Tyne Hospitals NHS Foundation Trust, Newcastle upon Tyne NE1 4LP, UK; anna.jackson25@nhs.net; 2 Translational and Clinical Research Institute, Newcastle University, Newcastle upon Tyne NE2 4HH, UK; m.p.cifuentes-nino2@newcastle.ac.uk

**Keywords:** necrotising enterocolitis, biomarker, point-of-care testing, urine, stool, saliva

## Abstract

Necrotising enterocolitis (NEC) remains an important disease for neonatologists, with diagnostic and management challenges and impacts on mortality and neurodisability. NEC can present in a non-specific way, and differentiating from late-onset sepsis (LOS), focal perforation (FIP) and feed intolerance can be difficult. Biomarkers have been extensively explored as a way to help more definitively identify NEC or rule it out. Many biomarkers that have been studied are blood biomarkers, and several other extensive reviews of biomarkers in NEC exist. In this narrative review, we focus on non-invasive samples, namely stool, urine and saliva, and on tests that are already available as point-of-care tests (POCTs) or are likely to be available as POCTs soon given current technologies. Faecal calprotectin and urinary intestinal fatty acid-binding protein (IFABP) have the most data to currently support their use in larger multi-centre studies and appear most likely to achieve translation into clinical practice. Saliva appears the most under-researched potential source of a non-invasive POCT for a biomarker for NEC. For faecal calprotectin and urinary IFABP, data that are most lacking relate to specificity, particularly the performance of these tests to differentiate NEC from FIP or LOS (occurring in the absence of NEC). We suggest a study design to facilitate moving towards the clinical use of non-invasive biomarkers in NEC.

## 1. Introduction

### 1.1. NEC

Necrotising enterocolitis (NEC) is a life-threatening bowel condition most commonly affecting preterm or very low-birth-weight (VLBW, <1500 g) infants. The precise aetiology is poorly understood but is underpinned by interactions between gut microbes and the host that precipitate exacerbated gut and systemic inflammation leading to necrosis [1]. Associated tight-junction breakdown increases the risk of bacterial translocation, meaning that around 10–20% of infants with NEC also have associated bacteraemia. Mortality rates are around 30–50% of infants with NEC, and there is significant neurodisability in survivors [2]. Current United Kingdom (UK) incidence reported by the National Neonatal Audit Programme (NNAP) showed that 5.1% of VLBW babies born in the UK in 2024 developed NEC [3]. Management includes antibiotics, usually stopping feeds, commencing parenteral nutrition (PN) and consideration for surgery [4]. An international study identified at least nine differing NEC definitions used in key publications, including Bells and the modified Bells criteria [5,6,7]. These mainly rely on clinical and radiological features (which are subjective); ‘routine’ blood results (platelet count, leucocytosis, pH, or c-reactive protein (CRP)) alone or in combination also contribute to some definitions or help classify severity [8], but problems with defining NEC are well-recognised [1]. There are no gold-standard diagnostic criteria for NEC. Intestinal symptoms that mimic NEC (abdominal distension, bilious aspirates and blood in stool) also occur with isolated late-onset sepsis (iLOS, bacteraemia without NEC), focal intestinal perforation (FIP) or feed intolerance. Treatments for FIP and iLOS differ from treatments for NEC, and improved discrimination of these conditions would benefit patients. Better discrimination would allow for better decision making around transfers to surgical centres [9], durations of ‘nil-by-mouth’ (NBM) and antibiotic duration. This would improve outcomes, facilitate better resource utilisation and reduce antimicrobial resistance. A good biomarker would increase NEC discrimination. This review seeks to address the specific complexities of biomarker use in NEC and review the existing literature in relation to non-invasive biomarkers to identify existing gaps in evidence and practice. We focus on point-of-care testing and suggest ways forward for the neonatal community. For practical applicability, we present potential biomarkers by non-invasive sample type.

### 1.2. Biomarkers

A biomarker can be defined as a measurable parameter that provides meaningful information regarding a specific clinical condition—here, NEC. Generic properties of good biomarkers include characteristics that relate to aetiology, with understanding of the timing of response. This allows for the interpretation of results and testing within appropriate time frames [10]. Biomarkers with early increase followed by decline with patient recovery allow for therapeutic monitoring and treatment adaption. Ideal biomarkers are sensitive (do not miss cases) and specific (do not confuse other pathologies for this specific disease) [11]. For preterm infants, an ideal NEC biomarker would also use a non-invasive sample (stool, urine or saliva rather than blood) and be undertaken on small-volume samples. Tests available as point-of-care tests facilitate rapid clinical decision making that improves outcomes [12]. For an NEC biomarker attempting to differentiate NEC from FIP or iLOS, cost is also important given the number of septic screens and episodes of abdominal concern experienced by this population [13]. It should also perform well across gestations, including the most immature (i.e., from 22 weeks), where aetiology and physiological responses may differ. It should perform well at varying postnatal ages and be independent of other intensive care processes and physiological adaptions that occur after birth. This includes performing well for those on parenteral or enteral nutrition [14]. Table 1 summarises properties of the ideal NEC biomarker.

Studies of potential biomarkers are common in medicine in general and within neonatology but are not always designed and reported optimally. The Standards for Reporting of Diagnostic Accuracy (STARD) Checklist for reporting diagnostic studies should be used for biomarker studies [15]. Studies should assess and demonstrate biomarker modification in diseased patients compared to controls, assess and report diagnostic properties of the biomarker, compare the diagnostic properties of the biomarker to existing tests, and demonstrate that the diagnostic properties of the biomarker improve physicians’ decision making. In addition, they should assess the overall usefulness of the biomarker, taking into consideration ease of use, invasiveness, timing of results, costs and consequences of therapeutic options. A successful biomarker study will show that use of the biomarker improves outcomes. For NEC, this may mean shortened time to surgery, reduced days NBM, or reducing the proportion of babies transferred to surgical centres who do not undergo surgery. Studies reporting all these elements for biomarkers are rare, and the appropriate statistical analysis is not always undertaken or presented. Thresholds for acceptable levels of sensitivity and specificity are not widely agreed or appropriately powered [16]. A recent systematic review (SR) of blood biomarkers in NEC used sensitivity and specificity values as high (>85%), moderate (70–85%) and low (<70%), with overall biomarker accuracy deemed high if both sensitivity and specificity exceeded 85%. This SR used values to determine the discriminative power of a biomarker based on Area Under Curve (AUC) values: poor (<0.5), moderate (>0.7), good (>0.8) and excellent (>0.9) [17]. Choice of cut-off values for receiver operator curves (ROCs) often does not acknowledge the grey zone [18], which lies between the areas of ‘exclusion with near-certainty’ and ‘diagnosis with near-certainty’ and is the area where additional testing may be clinically beneficial [19].

### 1.3. Specific Challenges in Biomarker Studies for NEC

The lack of a gold-standard test and the inability to always distinguish NEC from FIP or iLOS add additional difficulties to the search for an NEC biomarker [20,21]. Some studies include stage 1 NEC, whilst others exclude this; some classify NEC as ‘surgical’ or ‘medical’, and these variations make comparison difficult. NEC incidence also means that large sample sizes are needed but rarely undertaken in biomarker studies. Identified biomarkers need validation in larger populations before clinical use, and many potential biomarkers identified in the literature have not been tested further to facilitate their use in routine clinical practice [14]. For NEC, poor aetiological understanding, no gold-standard diagnosis and misclassifications within populations used in studies add to challenges in identifying and interpreting biomarker studies. Despite extensive publication of biomarker studies for NEC a standardised approach by clinicians to assessing, validating and using biomarkers in clinical practice has not yet been achieved. Although physiological and radiological biomarkers have been explored for NEC, such as heart rate variability [22], abdominal ultrasound [23] and near-infrared spectroscopy (NIRS) [24], this paper will focus on potential molecular biomarkers. Given the recent blood-focused biomarker SR by Molloy et al., we have limited our review to biomarkers using non-invasive samples—stool, urine and saliva, available or potentially adaptable as point-of-care tests (POCTs) [17].

## 2. Potential NEC Biomarkers

### 2.1. Faecal Biomarkers

Stool is generally easily obtained from the nappy with minimal disturbance to the infant. It may be limited in the first few days (when NEC is unusual) in infants that have never tolerated enteral feeds and in infants with paralytic ileus secondary to iLOS. Despite these limitations, many infants do stool regularly, and a successful faecal biomarker would be of clinical use.

#### 2.1.1. Faecal Proteins

Some faecal proteins, elevated by inflammation, are indicative of intestinal distress and the loss of barrier integrity that accompanies NEC. At present, several faecal proteins can be rapidly measured in stool and are (or are potentially) translatable into clinical practice by POCTs, discussed below.

#### 2.1.2. S100 Proteins

S100 proteins are a group of small calcium-binding proteins with both intra- and extracellular functions involved in inflammation [25,26].

#### 2.1.3. Calprotectin

Calprotectin is a heterodimer of S100A8 and S100A9 [25,26] released from activated granulocytes, particularly neutrophils, during inflammation [27] and in gut inflammation, and can be detected in stool. Faecal calprotectin (fCAL) is well-established in the diagnosis and monitoring of inflammatory bowel disease (IBD) [28]. Laboratory-based calprotectin testing is performed by enzyme-linked immunosorbent assay (ELISA) [29], which utilises antibodies to detect fCAL in stool. Validated POCTs are available utilising lateral-flow technology [30]. Normal levels of fCAL in preterm infants have been explored, are variable, and influenced by postnatal age, milk type [31,32,33,34] and corrected gestations [32,35]. A systematic review of articles analysing fCAL and NEC in preterm infants was published in 2016 [36], concluding that fCAL showed promise, but there were insufficient data to recommend clinical use. Ten years later, we identified 16 studies comparing fCAL in preterm infants with NEC against appropriate controls [37,38,39,40,41,42,43,44,45,46,47,48,49,50,51,52]. Although fCAL levels varied and many individual studies were small (six had <20 NEC cases), most (fourteen) studies identified statistically significant differences in NEC infants compared to controls (Table 2). Elevation both at clinical presentation and preceding this is described [53], and particularly high fCAL at diagnosis (>664.2 µg/g) was predictive of post-NEC stricture (sensitivity 86% and specificity 92%), supporting the concept that levels could be used to guide surgical decision making [54]. Whether fCAL can adequately discriminate NEC from iLOS or FIP is currently inadequately explored. One ‘NEC study’ above also included an iLOS population, showing that an fCAL level of 238 µg/g differentiated between NEC and iLOS [45]. Latif separately reports fCAL in a preterm population with iLOS, showing the utility of fCAL to discriminate between iLOS and healthy preterm infants (*p* < 0.001) [55]. The fCAL values for the iLOS group were median 94.56 (range 0.81–432.08)—units used are not clearly reported in this study, making comparison to NEC studies difficult, but the g/g values appear higher than in most reported NEC studies. Altamimi et al., in abstract form only, showed that fCAL levels were elevated in iLOS compared to healthy controls (average 320 µg/g vs. 190 µg/g), without further analysis [56]. One report identifies two cases of FIP where fCAL was lower than in three NEC cases (<2000 vs. >2000 µ/g) [57]. fCAL thus appears to have potential to act as a sensitive NEC biomarker and is available as a POCT, but differences in assay methodology, reporting units, gestational age, timing of sample collection, feeding status, and NEC severity or case definitions may contribute to the wide range of reported fCAL thresholds and limit the establishment of a universally applicable diagnostic threshold. There are currently inadequate data to confirm its ability to distinguish FIP and iLOS from NEC.

#### 2.1.4. S100A12

Faecal S100A12 (fS100A12, calgranulin C) is a calcium-binding antimicrobial protein used as a biomarker of intestinal inflammation in other populations [26] measured by ELISA. In eight cases of NEC at presentation and sixteen matched controls, a fS100A12 cut-off of 54.9 ng/mg had sensitivity 38% and specificity 94% [58]. In 145 VLBW infants (18 NEC), fS100A12 was significantly higher in severe NEC at onset and at 10 days before symptoms [59]. A cut-off value of 65 µg/g had sensitivity 0.76 and specificity 0.56, suggesting that fS100A12 may have a role in both predicting NEC and differentiating disease severity. However, significant inter- and intra-individual variability was reported. To the best of our knowledge, fS100A12 levels in iLOS or FIP have not been reported in the literature.

#### 2.1.5. Neutrophil Gelatinase-Associated Lipocalin (NGAL)

NGAL (lipocalin-2) is a protein produced by gut epithelial cells in response to inflammation, measured in stool by ELISA [60]. In eight stage 3 NEC and fourteen matched controls, faecal NGAL (fNGAL) was discriminatory up to 10 days before presentation, and when combined with fCAL, predicted 50% of cases of NEC [61]. We have not found literature exploring fNGAL in medical NEC, iLOS, FIP or feeding intolerance.

#### 2.1.6. Intestinal Fatty Acid-Binding Protein (I-FABP)

I-FABP is a protein exclusively synthesised in enterocytes, released on their death, and measurable by ELISA in serum, urine and stool [62]. In a recent case–control study of eight NEC infants (Stage ≥IIB), Avula et al. found that faecal I-FABP (fI-FABP) was significantly increased in NEC compared to controls at diagnosis (*p* < 0.05). At a cut-off of 0.36 ng/mg, sensitivity was 75% and specificity 94% with an AUC of 0.81 [58]. We have not identified further literature on fI-IFABP nor literature exploring stool I-FABP in iLOS, FIP or feeding intolerance.

#### 2.1.7. Keratin 8 (K8)

Keratin 8 is a cytoskeletal protein found in enterocytes, detected in stool by ELISA. In five infants with NEC, faecal K8 (fK8) was significantly increased from 48 h before diagnosis [63]; fK8 has not been further reported in NEC, nor in iLOS, feeding intolerance or FIP.

#### 2.1.8. Intestinal Alkaline Phosphatase (IAP)

IAP is an enzyme acting on the lipopolysaccharide (LPS) component of gram-negative bacterial cell walls. Faecal IAP (fIAP) activity increases upon initiation of the inflammatory cascade. In 136 preterm infants, 25 with severe NEC and 19 suspected, Heath et al. Showed that fIAP abundance and activity differentiated severe and suspected NEC from healthy infants (99% vs. 123% vs. 4.8%; AUC 0.97 for severe and suspected NEC). Twenty-six iLOS cases did not differ in fIAP measures from healthy infants, making IAP a potential early discriminator between NEC and iLOS, although causative organisms were not reported [64]. Unfortunately, others have not replicated this; Thibault et al. found no significant difference between fIAP measures between NEC and controls [61] but were underpowered (eight NEC) based on Heath’s data [64].

#### 2.1.9. Human Beta-Defensin-2 (HBD-2)

HBD-2 is an antimicrobial peptide quantifiable in stool via ELISA. Jenke et al. found that faecal HBD-2 (fHBD-2) increased in extremely low-birth-weight (ELBW) infants (<1000 g) with moderate (n = 6) but not severe NEC (n = 6) in the 72 h before diagnosis compared to controls [65]. It may therefore have a role in predicting severity, but not for diagnosis.

#### 2.1.10. Stool Proteomics

Proteomic technology is expanding the ability to identify faecal proteins as biomarkers for diseases, but current studies are limited and not suitable for POCTs. However, technological advances may change this, and pilot work suggests potential utility [66]. Metaproteomic analysis of stool is feasible, but the management of the data produced by this technology is challenging given the large number of proteins present [67,68].

#### 2.1.11. Surrogate Markers of Gut Microbiome

NEC is preceded by dysbiosis [69], but faecal microbiome changes are difficult to measure in a clinical setting, have high intra-individual variability, and depend on hospital sites [70]. Hooven et al. utilised machine learning with microbiome and clinical data generated from 45 NEC infants with 116 VLBW controls to develop a predictive model for NEC, predictive 24 h before disease onset (AUC 0.9) [71]. Dysbiosis-related measures may therefore be useful biomarkers of NEC, but a POCT of the microbiome is currently challenging. Stool metabolites represent metabolic interactions of the gut microbiome and host, and similar to microbiomic studies, stool metabolomics have been explored in NEC. Changes have been detected in amino acids and the tricarboxylic acid (TCA) cycle [72]. The amino acid profile of 31 NEC infants differed up to 3 days before NEC onset compared to controls [73]. Paired microbial and metabolomic profiling of 7 infants with NEC compared to 28 matched controls likewise identified metabolites of interest associated with NEC [74]. Infants with iLOS metabolite profiles have not been directly compared to NEC infants but have been assessed [75]. Faecal Volatile Organic Compounds (fVOCs) are volatile metabolites in stool and can be analysed using a handheld electronic nose (eNose) device to produce qualitative data or by gas chromatography–mass spectrometry (GC–MS) to produce quantitative results [76]. In small numbers of NEC cases, specific VOCs were absent in NEC up to 4 days prior to onset of symptoms [77]. De Meij et al. compared fVOCs in NEC, controls and iLOS from 5 days before to the day of diagnosis, showing NEC discrimination 2–3 days before disease onset, but not the day before or day of diagnosis [78]. In a multi-centre study, fVOCs were found to identify NEC but not in a way that could translate to clinical use [79]. More recently, Hassani et al. used GC–MS in 44 preterm NEC and matched controls. VOC analysis clearly distinguished cases and controls (AUC 0.82), with fourteen unique VOC features contributing to this [80]. In other settings, metabolites released by gut microbiota have been noted to induce epigenetic modifications in the host [81] and DNA methylation in NEC-associated genes (TLR4, VEGFA and DEFA5), which differed in NEC compared to controls [82]. As technology advances, ‘omic’ technologies, machine learning and artificial intelligence may support multi-omic POCTs of these proxy measures of microbe–host interactions that are not currently possible.

#### 2.1.12. Summary for Faecal Biomarkers

Currently, fCAL holds the most potential for clinical use as a point-of-care biomarker for NEC, differentiating NEC from controls, but with limited data to confirm whether iLOS and other ‘NEC-mimicking’ pathologies would be discriminated. More limited evidence suggests that fCAL may be useful for prognostication and has potential to guide management, including surgical decision making. The other stool biomarkers explored are reported in a small number of studies, each with limited numbers of NEC cases, and their ability to differentiate between iLOS and NEC is inadequately explored.

### 2.2. Urine Biomarkers

Urine can easily be obtained non-invasively using cotton wool in the nappy, although care is needed to avoid stool contamination [83]. Urine biomarkers may require normalisation with creatinine.

#### 2.2.1. Gut-Specific Proteins in Urine

Gut-specific proteins increase in urine with the loss of gut mucosal integrity that accompanies NEC [84].

Intestinal Fatty Acid-Binding Proteins (I-FABPs)

Fatty acid-binding proteins (FABPs) are a family of intracellular proteins with a variety of functions relating to transport, storage and metabolism of phospholipids. They have tissue-specific functions and are named after the organs in which they are expressed [85]. I-FABP is the most extensively explored urinary biomarker for NEC—enterocyte death results in the liberation of cytoplasmic I-FABP [62,86]. It is measurable in urine by ELISA or radioimmunoassay. Urinary I-FABP (uI-FABP) correlates with serum levels [87,88]. Studies assessing uI-FABP in NEC in comparison to preterm non-NEC controls are summarised in Table 3.

uI-FABP values were discriminatory in most studies [86,87,89,91,92,94,96,97,98,99]. Several studies showed a positive correlation between uI-FABP and NEC severity, in terms of staging [90,93,95] or other measures including resection length [101] or death as an outcome [86] (Table 3). However, not all studies support the utility of uI-FABP for either diagnosis [90,95] or staging/prognosticating [100]. Mannonia et al. measured uI-FABP within 90 h of birth, finding a significant difference between those who developed NEC and those who did not, suggesting changes may occur early [91]. Similarly, Gregory et al. found a significant difference in uI-FABP levels between NEC and controls 3 and 7 days before onset of symptoms [94]. Two studies explored whether uI-FABP could be useful in predicting response to treatment. Reisinger et al. found that higher urinary I-FABP levels were associated with unsuccessful reintroduction of enteral feeds following the NEC episode, and Kuik et al. found that higher uI-FABP levels were associated with the development of post-NEC strictures [93,102].

Unlike some other biomarkers, uI-FABP has been explored in iLOS and appears to discriminate between iLOS and NEC. Coufal et al. conducted an initial study identifying a significant difference in uI-FABP levels between NEC and LOS, substantiated in 2020 (AUC 0.831) [97,98]. A more recent study confirmed the utility of uI-FABP in a secondary analysis of the preterm neuroprotection trial, where infants with NEC had higher uI-FABP levels (median 11 vs. 2.6 ng/mL, *p*  =  0.006) [103]. Urinary I-FABP is therefore a promising non-invasive biomarker for NEC, differentiating both health and iLOS from NEC.

#### 2.2.2. Liver Fatty Acid-Binding Protein (L-FABP)

Coufal et al. also measured urinary L-FABP (uL-FABP) in their study (NEC (n = 20) or iLOS (n = nine), and eight healthy controls). Infants with NEC had significantly higher uL-FABP levels than healthy infants or those with iLOS [98]. We are not aware of other studies of uL-FABP.

#### 2.2.3. Trefoil Factor-3 (TFF-3)

TFF-3 is a peptide expressed in the intestinal tract with a role in maintaining mucosal barrier integrity. It is elevated in patients with IBD and can be measured by ELISA. Coufal et al. also measured urinary TFF-3 (uTFF-3) in their preterm case–control study discussed above. Infants with NEC had a significantly higher uTFF-3 level compared to control infants, but uTFF-3 did not distinguish between NEC and iLOS [98].

#### 2.2.4. Claudins

Claudins are tight-junction proteins found in intestinal epithelial cells helping maintain mucosal integrity. A study of six neonates, three with NEC, measured urinary claudin-2 (uclaudin-2) by Western blot longitudinally. In two NEC patients, uclaudin-2 was elevated at presentation, and uclaudin-2 differentiated NEC and control neonates (*p* = 0.05), and active NEC and NEC-free periods in NEC patients (*p* < 0.0001) [104]. Likewise, Thuijls et al. showed that uclaudin-3 differentiated confirmed from suspected NEC. A proposed cut-off of 800.8 INT gave a sensitivity of 71% and specificity of 81%, with an AUC of 0.76. Claudin-3 levels did not distinguish NEC stages [86].

#### 2.2.5. Caveolin

Caveolins are tight-junction proteins that act synergistically with claudins to maintain the gut mucosal barrier [105]. They can be measured in urine using an ELISA. A single-centre study of 12 preterm NEC infants and 12 healthy preterm controls measured urine caveolin-1 (ucaveolin-1) levels on day 3 and day 5 of life. Day 3 ucaveolin-1 ≥17.81 ng/dL predicted NEC with a sensitivity of 87.5% and specificity of 75.0% [106]. We have not found studies exploring ucaveolin-1 levels at the onset of NEC, in iLOS, feeding intolerance or FIP.

#### 2.2.6. Epidermal Growth Factor (EGF)

EGF is a peptide secreted into the intestinal lumen throughout the gastrointestinal tract and can be measured in urine (and saliva and serum) by ELISA. It has a role in preserving intestinal barrier function [107]. A study from 1991 showed that there was a significant increase in urinary EGF (uEGF) at diagnosis of NEC (n = 28) compared to healthy controls (n = 47) [108]. However, this was not replicated in a subsequent study; although salivary and serum EGF levels were different in NEC and controls, uEGF was not [109]. Urinary EGF has not been explored in LOS, FIP or feeding intolerance.

#### 2.2.7. Non-Specific Inflammatory Mediators in Urine

Serum Amyloid A (SAA)

Serum amyloid A is an acute-phase protein with a small molecular size, produced by the liver and kidneys in response to pro-inflammatory cytokines and can be measured in both serum and urine [100]. Reisinger et al. performed a retrospective study of 29 infants with NEC. Urine SAA (uSAA) was significantly higher in those with surgical NEC compared to medical NEC and stage II NEC compared to stage III NEC [100]. Coufal et al. found that uSAA levels significantly differed between NEC and controls, but also with iLOS, which uSAA did not differentiate from. When combined with I-FABP, the AUC for differentiating between NEC and controls was 0.941 [98]. Given its lack of specificity to gut mucosal insults, uSAA may have a role in severity prediction in NEC but may not be able to differentiate NEC and iLOS.

#### 2.2.8. Urinary Metabolomics

Urinary metabolites can be measured similarly to those in stool, but urine is a potentially easier sample to collect and work with, and the renal excretion of metabolites makes urinary metabolomics a potentially promising area for future biomarkers for NEC [83]. As noted earlier, metabolomics is generally not currently suitable for POCT but may identify suitable candidate molecules for the development of a POCT [110]. A urinary alanine:histidine ratio of >4 had a sensitivity of 82% and specificity of 75% for predicting NEC when taken between day four and nine of life [111]. Picaud et al. performed a pilot longitudinal study of 18 preterm infants using nuclear magnetic resonance (NMR) spectroscopy for metabolomic analysis of 96 urine samples. More complex differences were seen in urinary lactate, betaine, myo-inositol, urea, creatinine and N,N-dimethylglycine in infants who developed NEC compared to healthy infants [112]. Urinary tyrosine, arginine and riboflavin differentiated NEC from controls (AUC 0.963) in a case–control study using a combination of non-targeted NMR spectroscopy and targeted liquid chromatography MS of 108 metabolites [113]. However, the different metabolites identified in different studies suggests caution in clinical use of urinary metabolites as an NEC biomarker and may again raise the importance of the site and its influence on microbiome–host interactions. A recent critical appraisal of metabolomics in iLOS did not identify any studies seeking to discriminate iLOS from NEC [114].

#### 2.2.9. Urinary Proteomics

The urinary proteome contains >1500 different peptides. MS can identify a large number of different peptides and identify patterns of peptides in different disease processes. These peptides can then be validated using ELISA or other methods [115]. Sylvester et al. identified a 7-biomarker urinary panel with an AUC of 0.98 for differentiating NEC from iLOS and medical from surgical NEC (AUC of 0.984) in a multi-centre cohort [116]. A further prospective study identified three urinary fibrinogen peptide biomarkers, which produced an AUC of 0.856 for predicting surgical NEC [117]. More recently, Mackay et al. used aptamer-based proteomic technology in 20 infants with NEC matched to eight age-matched and 12 self-matched controls. Ninety-nine proteins significantly differed between NEC and controls. They developed two panels, one of intestinal inflammatory proteins (AUC of 0.9) and one that included proteins with the largest differences between NEC and controls (AUC of 0.98) [118].

#### 2.2.10. Summary for Urine Biomarkers

Currently, uI-FABP is promising in terms of an NEC biomarker as it can predict the onset of NEC and shows promise in terms of prognostication and guiding management decisions, and it appears to differentiate between NEC and iLOS. There is currently an available POCT for uLFABP but not IFABP, but technologically, a uIFABP POCT is achievable. As omic technologies advance, the development of POCTs may allow for a POCT of urinary metabolites to be used either as a direct biomarker of NEC, or to identify metabolites that could be selected for individual POCT assays.

### 2.3. Saliva

Saliva is routinely suctioned from preterm neonates for their comfort/care and also easily collected using specifically designed collection devices [119,120,121]. Salivary biomarkers are generally measured using ELISA. The COVID-19 pandemic promoted the production and use of POCTs on saliva through lateral-flow devices, with an acceleration of research and technology in this area [122,123].

#### 2.3.1. Salivary Markers of Healthy Gut Development

Salivary biomarkers may be of benefit in NEC; however, some are also found in breastmilk, and given the increase in the routine use of buccal colostrum, this may be problematic. Below, we explore current data on salivary biomarkers.

#### 2.3.2. Epidermal Growth Factor (EGF)

Epidermal growth factor (EGF) is found in multiple bodily fluids, the EGF receptor is found on enterocytes, and EGF has been implicated in NEC development [124]. Salivary EGF (sEGF) can be measured by ELISA. sEGF was significantly lower in 15 NEC infants at diagnosis compared to 12 controls (*p* < 0.001) [125], and in surgical NEC infants post-operatively compared to controls (*p* < 0.001) [109]. sEGF has also been shown to be lower in the first week of life in infants later developing NEC [126,127]. Studies measuring salivary EGF in iLOS or FIP are lacking.

#### 2.3.3. Salivary Inflammatory Markers

Salivary inflammatory markers have been explored in the context of neonatal sepsis but not NEC, and their potential is discussed below.

#### 2.3.4. Salivary C-Reactive Protein (CRP)

C-reactive protein (CRP) is an acute-phase reactant with clinical value in the serum in NEC prognostication and guiding surgical decision making [128]. In serum, it can be measured using ELISA, and POCTs are available. Salivary CRP (sCRP) correlates to serum and can discriminate a serum CRP of ≥10 mg/L in infants with mixed disease aetiology (including NEC and FIP) [129]. The POCT showed that sCRP had the same sensitivity (72%) and a higher specificity (89% vs. 70%) compared to serum CRP for detecting iLOS [130]. Given the current understanding, it is unlikely that sCRP would be specific for NEC, but it may help in differentiating feed intolerance from NEC or iLOS, allowing for early refeeding.

#### 2.3.5. Salivary Procalcitonin

Procalcitonin (PCT) is a pre-hormone produced by the thyroid gland with a role in calcium homeostasis; extra-thyroid synthesis occurs in response to iLOS [131]. In NEC without associated LOS, procalcitonin levels were elevated compared to healthy infants in one study [132], but in a second study, serum procalcitonin levels were comparable in NEC to healthy infants, and lower in NEC than in iLOS [133], and serum procalcitonin acted as a marker of NEC severity in a third study [134]. In infants below one year of age with iLOS, a significant correlation between salivary PCT (sPCT) and serum procalcitonin levels has been shown (r = 0.250, *p* = 0.001), and sPCT differentiated bacterial from viral infections [135]. There are no direct studies of sPCT in NEC, but sPCT appears useful to separate NEC from healthy infants. Further studies are required to identify whether sPCT separates NEC from iLOS or FIP.

#### 2.3.6. Salivary ‘-Omics’

There are limited published studies exploring salivary omic technology in NEC. The potential of newborn salivary proteomics in diagnosis has been recognised [136], and in ten infants with NEC, lower amounts of interleukin-1 receptor antagonist were detected in saliva in the two to three weeks before onset compared to controls [137]. Characteristic cytokine profiles have been identified in other bodily fluids [138] associated with NEC, and salivary proteomics may have potential to detect cytokines non-invasively [139], but this has not currently been studied.

Transcriptomics measures RNA transcripts produced by the genome and is a marker of gene expression [140]. Maron et al. have studied the salivary transcriptome of healthy preterm infants and established a small (five preterm infants) control cohort that could be utilised to identify gene expression changes in conditions such as NEC [141,142,143]. This work of Maron has formed the foundation of neonatal salivary omic research; the next steps require comparing preterm proteomes and transcriptomes from healthy infants to those with NEC, iLOS and FIP.

#### 2.3.7. Summary for Saliva

Salivary biomarkers for NEC are relatively underexplored compared to other biological fluids and have considerable potential in terms of the development of non-invasive POCTs but are currently not in a position to directly translate to clinical use.

## 3. Discussion

NEC is a heavily researched and much discussed neonatal topic, and the difficulties in diagnosis are well-established. Accurate diagnosis matters for each baby and for any research where NEC or its potential mimickers are outcomes. A biomarker that facilitated early diagnosis or allowed for the early exclusion of a diagnosis of NEC would be clinically important. NEC biomarkers are heavily researched, most commonly in the blood, and we have focused here on biomarkers in non-invasive samples, as well as those already available as POCTs or potentially modifiable to a POCT technology. Despite the extensive literature, many studies are of small numbers of NEC cases, and variation in methodology, reporting units, sample timing and case selection make interpretation limited. However, of these, the most well-studied and promising faecal biomarker to date is fCAL, already available as a POCT, where extensive data suggest that fCAL is sensitive for NEC, but specificity, and especially the ability to differentiate NEC from iLOS or FIP, requires further work. The most promising urine biomarker to date is uI-FABP, which appears moderately sensitive and specific, although the number of infants studied to date remains small, and a current POCT is not available, although a POCT for uL-FABP is available, making a POCT possible for uI-FABP. Salivary NEC biomarkers are the least well-explored and only offer research potential at this stage.

## 4. Conclusions and Future Work

**Figure 1:**
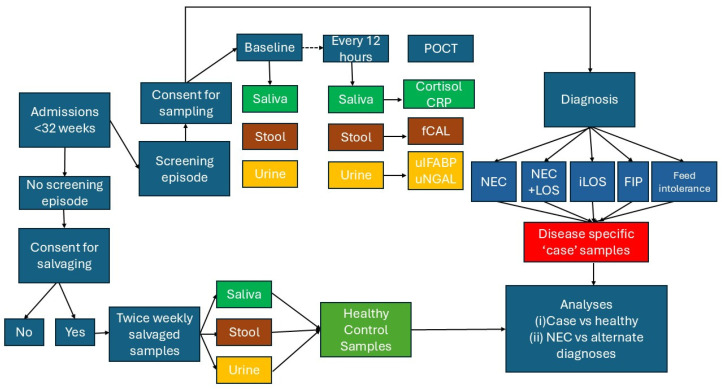
Suggested design for POCT biomarker study. POCT = point-of-care test, NEC = necrotising enterocolitis, iLOS = isolated late-onset sepsis, FIP = focal intestinal perforation, LOS = late-onset sepsis, fCAL = faecal calprotectin, CRP = C reactive protein, uIFABP = urinary intestinal fatty acid-binding protein, uNGAL = urinary neutrophil gelatinase-associated lipocalin.

Perhaps the most surprising aspect of biomarkers for NEC is that despite promising non-invasive biomarkers for NEC diagnosis and prognostication, none have yet translated into clinical practice. This may relate to ongoing uncertainty about which infants have been included in studies to date, the lack of a gold-standard diagnosis, the single-site nature of many studies, and the small size of individual studies. We propose the ‘ideal’ NEC biomarker study for these non-invasive biomarkers in Figure 1.

This involves the consentual salvaging of samples already taken for clinical reasons to obtain ‘control’ samples, as described in our SERVIS (supporting enhanced research in vulnerable infants) [144] and suggests separately consenting infants undergo additional sampling when being screened for possible infection, NEC or feed intolerance. This would ideally be undertaken in multiple centres, use actual POCTs and leave the clinical team unaware of the results. Performance, measured by sensitivities and specificities of individual assays, and combinations of assays could then be evaluated across centres explored, and potential clinical roll out may result. Once in use clinically, these POCTs could be additionally evaluated to ensure meaningful clinical impact on families and babies, as well as on services, including cost effectiveness. However, given the technological advances in POCT testing brought about by the COVID pandemic and the huge cost to health services of NEC, it is highly likely that a POCT could be made available that would be cost-effective. These could be measured by reduced time to diagnosis, faster transfer of babies who need surgery, reduced transfer of babies who never need surgery, impact on time spent NBM and on antibiotics, and potential other measures. Although several non-invasive biomarkers show promise, further large-scale multi-centre validation studies are required before routine clinical implementation can be achieved. For NEC biomarkers, we are not quite ‘there’ but could be soon.

## Figures and Tables

**Table 1:** Properties of the ideal NEC biomarker.

Property	Required Characteristic	Importance
Diagnostic Accuracy	High Sensitivity >85%	Ensures all cases are identified, allowing for early management and transfer to appropriate centre.
	High Specificity >85%	Capable of distinguishing NEC from iLOS or FIP as clinical management differs; important for research where NEC, iLOS or FIP are outcomes and correct categorisation of disease crucial.
Kinetics	Increases early	Increase detectable before or at clinical onset, i.e., before the diagnosis is achieved by other means.
	Rapid Response to clinical improvement	Should decline quickly when successful treatment is occurring; allows infants not at risk of needing surgery to avoid transfer, minimises time spent NBM and/or on antibiotics.
Clinical Utility	Non-invasive sample—urine, stool, saliva	Acceptable to families, most easily accessible (stool may not always be).
	Ease of use, rapid result <2 h	Ideally available as a point-of-care test (POCT) or rapid ability to see translation to POCT in the future. Allows decision making on transfer, feeding, surgery.
	Reliable threshold values	Well-defined cut-offs in well-designed studies, ideally validated for each (or small groups of) gestational ages, in multi-centre studies, and in infants that are enterally fed and on parenteral nutrition.
	Improves outcomes	Shown to improve clinical outcomes of value to families and clinicians.
Predictive Value	Severity correlation	Differentiates between medical NEC and surgical NEC.
	Prognostic ability	Capable of predicting long-term outcomes of value to families and clinicians.

**Table 2:** Case–control studies of fCAL in preterm NEC vs. healthy controls.

fCAL Studies of NEC vs. Control in Preterm Infants						
Study (Author, Year)	Stages	NEC (n) vs. Healthy (n)	Gestational Age (Weeks)	Results NEC:No NEC	*p*-Value	Cut-Off (µg/g), Sensitivity%/Specificity%
Abdelkader 2019 [37]	I–III	29 vs. 29	33.8 (±1.3) vs. 34.1 (±1.2)	453.6 (±259.6):65.3 (±24.9)	<0.001	153, 96.6/100
Albanna 2014 [38]	I–III	15 vs. 20	32.3 (±2.3)	241.1 (±57.5):72.6 (±10.6) [mg/dL]	<0.001	Not given
G Aydemir 2013 * [39]	I–III	10 vs. 9	29 (28–30) vs. 29 (28–30)	185 (165–198):104 (92–123)	0.006	Not given
O Aydemir 2012 [40]	II–III	25 vs. 25	28.3 (±2.5) vs. 28.9 (±2)	1282 (241–3337):365 (58–1006)	<0.001	792, 76/92 AUC 0.89
Barekatain 2019 [41]	II–III	35 vs. 35	29.41 (+/−2.2) vs. 28.99 (+/−1.28)	4460 (±172.6):103 (+ 31)	<0.001	176, 97.14/100
Bin-Nun 2015 [42] Quantum blue	II–III	13 vs. 16	27.9 (±2.4) vs. 28.8 (±1.8)	3000 (2075–7875):195 (110–440)	<0.001	480, 100/84.6
Carroll 2003 [43]	I–III	7 vs. 7	30 (±4) vs. 30 (±4)	288.4 (±49.1):98 (±60.6) [mg/L]	0.0006	Not given
Eskander 2020 [44]	I–III	60 vs. 40	<35	167 (116–2510):32.15 (25–90)	0.000	109.5, AUC 1
* Hong 2023 [45]	II–III	20 vs. 174	30.6 (±1.2) vs. (30 ±1.7)	2536 (1020–6230):not given	<0.001	1237, 93/75 AUC 0.83
Liu 2024 [46]	II–III	30 vs. 40	29.18 (±1.63) vs. 29.52 (±1.46)	521.6 (113.6–4358.2):213.3 (32.2–1036.3)	0.000	428.99, 76.7/67.5
MacQueen 2016 [47]	II–III	15 vs. 15	29.5 (±4.1) vs. 29.2 (±4/45)	516 (226–797):110 (39–165)	0.0001	AUC 0.942
Shalaby 2025 [48]	I–III	54 vs. 42	31.28 (±2.39) vs. 30.81 (±2.34)	362.6 (±239.8):61.9 (±44.2)	<0.001	176 87/97.6
van Zoonen 2018 [49]	II–III	10 vs. 30	27.5 (24.6–29.4) vs. 27.6 (25.3–29.9)	465 (160–620):390 (<40–2630)	0.8	N/A
Yoon 2014 [50]	I–III	4 vs. 12	29.4 (±3.5) vs. 29 (±3.1)	5.75 (±1.98):4.34 (±1.94) [mg/kg]	0.02	Not given
Zhang 2016 [51]	I–III	17 vs. 23	34.58 (±1) vs. 35.08 (±0.79)	858 (347.5–1417.5):179 (125–265)	<0.001	281, 88/83 AUC 0.931
ZongLi 2025 [52] chromatography	I–III	84 vs. 168	32 (±2.61) vs. 32.66 (±2.54)	31.9 (65.7–197.2):not given	<0.001	8.40, 43/89 AUC 0.65

Calprotectin is µg/g median (IQR) or mean (±SD) unless otherwise stated, analysis is by ELISA unless otherwise stated, AUC = Area under the curve. * 238 µg/g for NEC vs. iLOS (n = 34), sens 83%/spec 83%.

**Table 3:** Studies of urinary I-FABP.

Case–Control Studies of uIFABP					
Study Author, Year	Study Design	NEC (n) vs. Control (n)	Results NEC:Control (*p*)	Cut-Off	Sensitivity%/Specificity%
Derikx et al. 2007 [89]	NEC vs. non-NEC	5 vs. 12	3.9:1.2 (0.001)	2	Not given
Evennett et al. 2010 [90]	NEC vs. healthy	16 vs. 6	2.1 pg/mL:<1 pg/mL (0.055)	2	58/100 AUC 0.77
Thuijls et al. 2010 [86]	NEC vs suspected NEC	14 vs. 21	Not given	2.20	PLR 9.3, NLR 0.08
Mannoia et al. 2011 [91] first 90 h of life	NEC vs. non-NEC	17 vs. 38	Not given	>800 pg/mL	100/100
Reisinger et al. 2012 [92]	NEC vs. healthy	29 vs. 33	11.4:1.2 (<0.001)	2.4	79/85 AUC 0.88
Reisinger et al. 2013 [93] at refeeding	NEC vs. healthy	21 vs. 20	Not given/434 pg/mL	NS	
Gregory et al. 2014 [94] 7 days before onset	NEC vs. healthy	70 vs. 70	OR for 10-fold rise 4.14 (2.2–7.8) (<0.01)	T-3 13.9 ng/mL T-7 13.3 ng/mL	T-3 65/84 T-7 60/78
Schurink et al. 2015 [95]	NEC vs. suspected NEC	22 vs. 15	491 ng/mL:26 (0.19)	218 ng/mL	57/89
Gollin et al. 2014 [96]	NEC vs. healthy	5 vs. 21	Not given	10.2 pg/nmol	100/95.6
Coufal et al. 2016 [97]	NEC vs. iLOS vs. healthy	24 vs. 18 vs. 12	Not given	4.1 NEC:iLOS 2.52 NEC:controls	NEC vs. Sepsis 81/100 NEC vs. Controls 81/100
Coufal et al. 2020 [98]	NEC vs. iLOS vs. healthy	20 vs. 9 vs. 8	Not given	Not given	NEC vs. Sepsis AUC 0.831 NEC vs. control AUC 0.941 uiAFB + SAA, NEC vs. Controls AUC 0.941
El-Abd Ahmed et al. 2020 [99]	NEC vs. healthy	55 vs. 23 Controls	1.74 ng/g:0.6 (0.000)	2.93 ng/g creatinine	90/72 AUC 0.864
Shaaban et al. 2021 [87]	NEC vs. healthy	40 vs. 40	5009.22:2677.62 pg/mL, (0.04)	Not given	AUC 0.81
Severity studies					
Evennett 2010 [90]	Focal vs. Severe (multifocal or pan-intestinal)	6 vs. 6	1.1 pg/mmol vs. 7.4 pg/mmol	N/A	
Reisinger et al. 2013 [93]	poor outcome vs. good outcome	5 vs. 16	6: 0.38 (<0.01)	963 pg/mL	80/94
Reisinger et al. 2014 [100]	Stage predictor	13 Stage II vs. 16 Stage III	4800 vs. 11,100: NS	34.4	83/83
Schurink et al. 2015 [95]	Complex NEC vs. NEC	11 vs. 11	Not given	232 ng/mL	71/80
Heida et al. 2015 [101]	Severity predictor	19 surgical NEC	N/A	N/A	Correlation with length resected Rho = 0.92 (*p* = 0.001)

Results are pg/mol/Cr unless otherwise stated, cut-offs are Pg/nmolCr unless otherwise stated, all studies used ELISA, samples were at presentation unless otherwise specified, AUC = area under the curve, PLR = positive likelihood ratio, NLR = negative likelihood ratio, T-3 = 3 days before onset, T-7 = 7 days before onset, NA = not available

## Data Availability

Not applicable.

## Abbreviations

The following abbreviations are used in this manuscript:

AUC	Area under the curve
CAL	Calprotectin
CRP	C-reactive protein
EGF	Epidermal growth factor
f	Faecal
ELISA	Enzyme-linked immunosorbent assay
FIP	Focal intestinal perforation
GM–CS	Gas chromatography–mass spectrometry
HBD-2	Human beta-defensin-2
IAP	Intestinal alkaline phosphatase
IBD	Inflammatory bowel disease
I-FABP	Intestinal fatty acid-binding protein
iLOS	Isolated late-onset sepsis
K8	Keratin 8
LOS	Late-onset sepsis
IQR	Interquartile range
NBM	Nil by mouth
NMR	Nuclear magnetic resonance
NEC	Necrotising enterocolitis
NIRS	Near-infrared spectroscopy
NNAP	National neonatal audit programme
NGAL	Neutrophil gelatinase-associated lipocalin
PLR	Positive likelihood ratio
NLR	Negative likelihood ratio
PN	Parenteral nutrition
PCT	Procalcitonin
POCT	Point-of-care test
ROC	Receiver operator curve
s	Salivary
SAA	Serum amyloid A
SERVIS	Supporting enhanced research in vulnerable infants
STARD	Standards for Reporting of Diagnostic Accuracy
SR	Systematic review
TCA	Tricarboxylic acid
TFF3	Trefoil factor-3
u	Urinary
UK	United Kingdom
VLBW	Very low birthweight
VOCS	Volatile organic compounds
